# An insight to HTLV-1-associated myelopathy/tropical spastic paraparesis (HAM/TSP) pathogenesis; evidence from high-throughput data integration and meta-analysis

**DOI:** 10.1186/s12977-019-0508-8

**Published:** 2019-12-30

**Authors:** Sayed-Hamidreza Mozhgani, Mehran Piran, Mohadeseh Zarei-Ghobadi, Mohieddin Jafari, Seyed-Mohammad Jazayeri, Talat Mokhtari-Azad, Majid Teymoori-Rad, Narges Valizadeh, Hamid Farajifard, Mehdi Mirzaie, Azam Khamseh, Houshang Rafatpanah, Seyed-Abdolrahim Rezaee, Mehdi Norouzi

**Affiliations:** 1 Department of Microbiology, School of Medicine, Alborz University of Medical Sciences, Karaj, Iran; 2 Non-communicable Diseases Research Center, Alborz University of Medical Sciences, Karaj, Iran; 3 Instituto Gulbenkian de Ciência – IGC, Rua da Quinta Grande, 6, 2780-156 Oeiras, Portugal; 4 Department of Virology, School of Public Health, Tehran University of Medical Sciences, Tehran, Iran; 5 Institute of Biochemistry and Biophysics, University of Tehran, Tehran, Iran; 6 Research Program in Systems Oncology, Faculty of Medicine, University of Helsinki, Helsinki, Finland; 7 Research Center for Clinical Virology, Tehran University of Medical Sciences, Tehran, Iran; 8 Immunology Research Center, Inflammation and Inflammatory Diseases Division, Mashhad University of Medical Sciences, Mashhad, Iran; 9 Immunology-Microbiology Department, School of Medicine, Qom University of Medical Sciences, 14155-6447 Qom, Iran; 10 Department of Applied Mathematics, Faculty of Mathematical Sciences, Tarbiat Modares University, Tehran, Iran; 11 Pediatric Cell Therapy Research Center, Children Medical Center Hospital, Tehran University of Medical Sciences, Tehran, Iran

**Keywords:** HTLV-1, HTLV-1-associated myelopathy/tropical spastic paraparesis, HAM/TSP, High-throughput data integration, Meta-analysis, Microarray

## Abstract

**Background:**

Human T-lymphotropic virus 1-associated myelopathy/tropical spastic paraparesis (HAM/TSP) is a progressive disease of the central nervous system that significantly affected spinal cord, nevertheless, the pathogenesis pathway and reliable biomarkers have not been well determined. This study aimed to employ high throughput meta-analysis to find major genes that are possibly involved in the pathogenesis of HAM/TSP.

**Results:**

High-throughput statistical analyses identified 832, 49, and 22 differentially expressed genes for normal vs. ACs, normal vs. HAM/TSP, and ACs vs. HAM/TSP groups, respectively. The protein–protein interactions between DEGs were identified in STRING and further network analyses highlighted 24 and 6 hub genes for normal vs. HAM/TSP and ACs vs. HAM/TSP groups, respectively. Moreover, four biologically meaningful modules including 251 genes were identified for normal vs. ACs. Biological network analyses indicated the involvement of hub genes in many vital pathways like JAK-STAT signaling pathway, interferon, Interleukins, and immune pathways in the normal vs. HAM/TSP group and Metabolism of RNA, Viral mRNA Translation, Human T cell leukemia virus 1 infection, and Cell cycle in the normal vs. ACs group. Moreover, three major genes including STAT1, TAP1, and PSMB8 were identified by network analysis. Real-time PCR revealed the meaningful down-regulation of STAT1 in HAM/TSP samples than AC and normal samples (*P *= 0.01 and *P *= 0.02, respectively), up-regulation of PSMB8 in HAM/TSP samples than AC and normal samples (*P *= 0.04 and *P *= 0.01, respectively), and down-regulation of TAP1 in HAM/TSP samples than those in AC and normal samples (*P *= 0.008 and *P *= 0.02, respectively). No significant difference was found among three groups in terms of the percentage of T helper and cytotoxic T lymphocytes (*P *= 0.55 and *P *= 0.12).

**Conclusions:**

High-throughput data integration disclosed novel hub genes involved in important pathways in virus infection and immune systems. The comprehensive studies are needed to improve our knowledge about the pathogenesis pathways and also biomarkers of complex diseases.

## Background

HTLV-associated myelopathy/tropical spastic paraparesis (HAM/TSP) is a chronic neurodegenerative disease with progressive characteristics that disturbs the functioning of the sensory and motor nerves [1]. Indeed, infection with HTLV-1 can lead to asymptomatic carrier (AC) state or two diseases including Adult T-Cell Leukemia Lymphoma (ATLL) or/and HAM/TSP [2].

About 10–20 million people worldwide have been infected with HTLV-1 [3]. Endemic areas include the Middle East, Japan, the Caribbean basin, Central Africa, the Melanesian Islands, and South America. Only 2–5% of those infected with the virus develop HAM/TSP [4, 5].

Patients with HAM/TSP often have symptoms such as back pain, stiffness, and pain in the lower limbs, urinary frequency, and progressive weakness. Mild cognitive impairment is also common. The clinical signs of the disease imitate multiple sclerosis when the spinal cord is involved, such that sick people need walking aids after 1 year of illness [6].

HTLV-1 may weaken or impair the immune system, which results in autoimmunity to neurons. It also provides an immunosuppressive microenvironment that authorizes the HTLV-1 infected cells to escape host immune response and causes HTLV-1-associated diseases [7].

Studies on HTLV-1 as a factor that deregulates the host’s immune system has lasted for many years and has sometimes yielded polemical results. Despite various studies on how to treat HAM/TSP, it is still a challenge for clinicians [8–12]. Therefore, identifying prognostic biomarkers that implicated in the pathogenesis is vital to understand the development and progression of a disease, as well as its diagnosis and treatment. Since now, different genes that are involved in mTOR, NF-kappa B, PI3K, and MAPK signaling pathways have been known in the HAM/TSP cases. Also, apoptosis can occur in the cell nucleus of the HAM/TSP patients [2, 13, 14].

Microarray technology can simultaneously measure tens of thousands of genes from different tissue samples in a high-throughput and cost-effective manner [15]. However, the results may be irreproducible [16] or be influenced by the data perturbations [17, 18]. One possible solution to find robust information is the integration of multiple datasets which is called meta-analysis [19–22]. To this end, various statistical procedures are employed to combine and analyze the results of the independent studies. Meta-analysis increases the validity of the results and makes the possible estimation of gene expression differences [23].

In this study, we integrated 16 datasets in three groups to find gene signatures by network analysis of differentially expressed genes. The results specified the genes and pathways, which possibly have critical roles in the development of the HAM/TSP pathogenesis. Flow cytometry was employed to determine the ratio of CD4+ to CD8+ and better understanding the pathogenesis of the virus. Moreover, the real-time PCR confirmed different expressions of the determined genes in the HAM/TSP cases versus AC and normal subjects.

## Methods

### Database searching and identification of eligible studies

We searched the Gene Expression Omnibus (http://www.ncbi.nlm.nih.gov/geo/) and ArrayExpress (https://www.ebi.ac.uk/arrayexpress/) by end of 2018 to find datasets reporting the expression levels of miRNA and mRNA in the HAM/TSP and AC subjects. To find the relevant reports, keywords including Human T-lymphotropic virus 1-associated myelopathy/tropical spastic paraparesis, HTLV-1, TSP, HAM/TSP, asymptomatic carrier, AC, ACs were firstly used. The inclusion criteria were then research and regular studies that performed the high-throughput microarray studies on the human subjects. The normal samples were also considered to compare with these groups. The exclusion criteria were studies performed on the non-human samples, cell line, and non-blood samples. Moreover, two independent investigators searched and gathered data from each included study. The quality and consistency of the studies were evaluated by the R package MetaQC (0.1.13) [24]. Finally, the obtained data were classified into three groups named as ACs vs. normal, HAM/TSP vs. normal, and HAM/TSP vs. ACs.

### Pre-processing and meta-analysis

The expression data in each group were background corrected and quantile normalized using the Affy package implemented in R programming language (3.6.1) (http://www.r-project.org). The datasets were integrated individually at miRNA and mRNA levels using random effect method (REM) and then differentially expressed miRNAs (DEMs) and differentially expressed genes (DEGs) were identified by the R package MetaDE (1.0.5), respectively. The low number of DEGs caused that the p-values of less than 0.005 and logFC > |1| were further considered as a significant difference to have more DEGs and networks construction. The experimentally validated targets of each DEMs were obtained using miRTarBase (http://miRTarBase.cuhk.edu.cn/) [25] and then integrated super-horizontally with DEGs. The common genes were considered for further analysis.

### Networks construction

To construct the network comprises protein–protein interactions (PPIs) in each group, the STRING database version 11.0 was employed [26]. Seven interaction sources including physical interactions, functional association, high-throughput experiments, genomic context, co-expression, databases, and text-mining were considered. Then, the PPIs networks were analyzed in terms of degree by NetworkAnalyzer in Cytoscape 3.7.1. The degree is defined as the number of edges connected to a node [27]. The genes with higher aforementioned criteria were considered as hub genes.

### Module finding and pathways analysis

The ACs vs normal network clustering was implemented using the fast unfolding clustering algorithm in Gephi (0.9.2) [2, 28, 29]. The biologically meaningful modules were then chosen. The networks and modules were visualized by Cytoscape (3.7.1). To find the meaningful pathways in which hub genes are involved, g:Profiler web tool (version: 1185_e69_eg16) was employed [30]. The overall expressed gene lists for each group were considered as the background. Ten top pathway terms with higher P-value were selected for further interpretations.

### Patient population and sample collection

The blood samples were collected from eight patients with ACs, eight patients with HAM/TSP, and eight normal samples who referred to the neurology department of Ghaem Hospital, Mashhad University of Medical Sciences (MUMS). All specimens were collected after acquiring informed consent from the patient’s guardians. Two trained neurologists affirmed the diagnosis of HAM/TSP according to WHO criteria. All contributors had seropositive test for HTLV-1 by enzyme-linked immunosorbent assay (ELISA, Diapro, Italy). The results of serology were confirmed by PCR [31]. The participants had no history of treatment with IFNs. This study was approved by the Ethics Committee of Biomedical Research at TUMS (IR.TUMS.SPH.REC.1396.242).

### Flow cytometry analysis

To determine T helper and cytotoxic cells populations in HAM/TSP, ACs and normal groups; PerCP anti CD3 antibody (bio legend company cat no: 344813), Phicoerythrin (PE) anti CD4 antibody (bio legend company cat no: 317409) and PE anti CD8 antibody (bio legend company cat no: 301007) were used. Fresh peripheral blood samples were treated by lysis buffer for destroying the red blood cells and platelets. Samples were analyzed on a FACS caliber Becton Dichinson. All analyses were done in the lymphocyte gate.

### HTLV-1 proviral Load

Peripheral blood mononuclear cells (PBMCs) were isolated from EDTA-treated blood samples using Ficoll density gradient medium (Cedarlane, Hornsby, ON, Canada). The commercial blood mini kit (Qiagen, Germany) was applied to extract DNA from PBMCs. In order to measure the PVL of HTLV-I in PBMCs, a real-time PCR using a commercial real-time-based absolute quantification kit (HTLV-1 RG; Novin Gene, Karaj, Iran) was performed [32].

### Quantitative real-time PCR

Total RNA was extracted from fresh PBMCs using TriPure isolation reagent (Roche, Germany) according to the manufacturer’s instructions. Double-stranded cDNA was synthesized using the RevertAid TM first-strand cDNA synthesis kit (Fermentas, Germany). Following primers and probes were designed and used to determine the expression levels of STAT1, PSMB8, TAP1 : STAT1 (forward primer: 5ʹ-AACATGGAGGAGTCCACCAATG-3ʹ, reverse primer: 5ʹ-GATCACCACAACGGGCAGAG-3ʹ and TaqMan probe: FAM- TCTGGCGGCTGAATTTCGGCACCT -BHQ1), PSMB8 (forward primer: 5ʹ-GTTCAGATTGAGATGGCCCATG-3ʹ, reverse primer: 5ʹ-CGTTCTCCATTTCGCAGATAGTAC-3ʹ and TaqMan probe: FAM- CCACCACGCTCGCCTTCAAGTTCC -BHQ1), TAP1 (forward primer: 5ʹ-TACCGCCTTCGTTGTCAGTTATG-3ʹ, reverse primer: 5ʹ-GAGCCCAGGCAGCCTAGAAG-3ʹ and TaqMan probe: Fam-CGCACAGGGTTTCCAGAGCCGCC-BHQ1). The primers and probes of Tax and HBZ were synthesized according to published data [33]. The relative 2 standard curves real-time PCR was carried out on the cDNA samples using TaqMan master mix (Takara, Otsu, Japan) and a Q-6000 machine (Qiagen, Germany). The GAPDH gene was employed as a housekeeping gene to normalize the mRNA expression levels, and also to control the error between samples [32, 34].

### Statistical analysis

Statistical analysis was carried out using GraphPad Prism Software Version 7 (GraphPad software, Inc). Quantitative data were expressed as mean ± SEM and percentages. The comparisons between various groups were accomplished using ANOVA. Pearson’s or Spearman’s tests were used for the analysis of the correlation between variables. The outcomes were considered significant if *P* ≤ 0.05.

## Results

### Studies included in the meta-analysis

According to our inclusion/exclusion criteria, 16 studies were found in the GEO repository datasets which were performed at mRNA or miRNA levels. After quality control done by MetaQC package, seven (GSE29312 [35], GSE29332 [35], GSE46518 [36], GSE52244 [37], GSE55851 [38], GSE11577 [39], GSE46345 [36], three (GSE19080, GSE29312, GSE29332), and four (GSE38537 [40], GSE29312, GSE29332, GSE19080) mRNA and miRNA datasets were of high quality for further analyses of normal vs. ACs, normal vs. HAM/TSP, and ACs vs. HAM/TSP groups, respectively (Table 1).

**Table 1:** Selected studies included in the meta-analysis

Row	Normal vs. ACs	Type	Tissue	Number of samples
1	GSE29312	Expression profiling by array	Whole Blood	Normal: 9, ACs: 20
2	GSE29332	Expression profiling by array	Whole Blood	Normal: 8, ACs: 17
3	GSE46518	Expression profiling by array	CD4+	Normal: 6, ACs: 6
4	GSE52244	Expression profiling by array	CD4+	Normal: 3, ACs: 3
5	GSE55851	Expression profiling by array	CD4+	Normal: 3, ACs: 6
6	GSE11577	Non-coding RNA profiling by array	PBMCs	Normal: 3, ACs: 4
7	GSE46345	Non-coding RNA profiling by array	CD4 + and CD8 +	Normal: 12, ACs: 12

Row	Normal vs. TSP	Type	Tissue	Number of samples
1	GSE19080	Expression profiling by array	CD4+	Normal: 8, HAM/TSP: 12
2	GSE29312	Expression profiling by array	Whole Blood	Normal: 9, HAM/TSP: 10
3	GSE29332	Expression profiling by array	Whole Blood	Normal: 8, HAM/TSP: 10

Row	ACs vs. TSP	Type	Tissue	Number of samples
1	GSE38537	Expression profiling by array	CD4+	ACs: 4, HAM/TSP: 4
2	GSE29312	Expression profiling by array	Whole Blood	ACs: 20, HAM/TSP: 10
3	GSE29332	Expression profiling by array	Whole Blood	ACs: 17, HAM/TSP: 10
4	GSE19080	Expression profiling by array	CD4+	ACs: 11, HAM/TSP: 12

### Differentially expressed genes and miRNAs

A total of four miRNAs including hsa-mir-218, hsa-mir-206, hsa-mir-31, and hsa-mir-34A were identified as DEMs between normal and AC group. The target genes of the mentioned DEMs were further identified in miRTarBase. A total of 663 genes were identified as target and added to 180 DEGs obtained across microarray datasets. After removing duplicate genes, 832 DEGs were specified. Also, a total of 49 and 22 genes were identified as DEGs for normal vs. HAM/TSP and ACs vs. HAM/TSP groups, respectively (Additional file 1: Table S1).

### Protein-protein interactions networks (PPINs) and Module finding

**Fig. 1:**
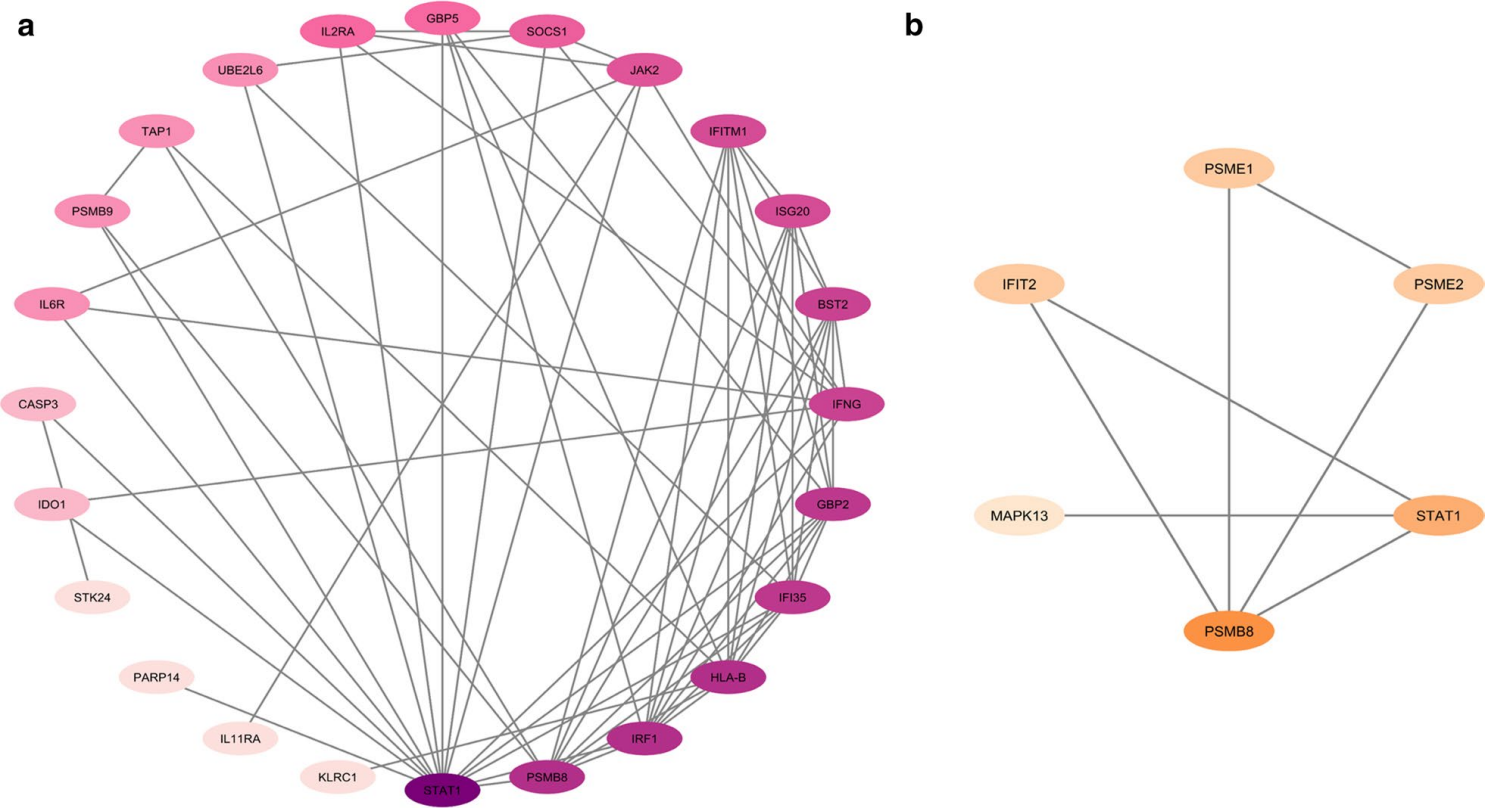
The PPINs constituted between the identified hub DEGs of **a** Normal vs. HAM/TSP and **b** ACs vs. HAM/TSP groups. The color is indicative of the degree level, so that bold color indicates the higher degree of node

**Fig. 2:**
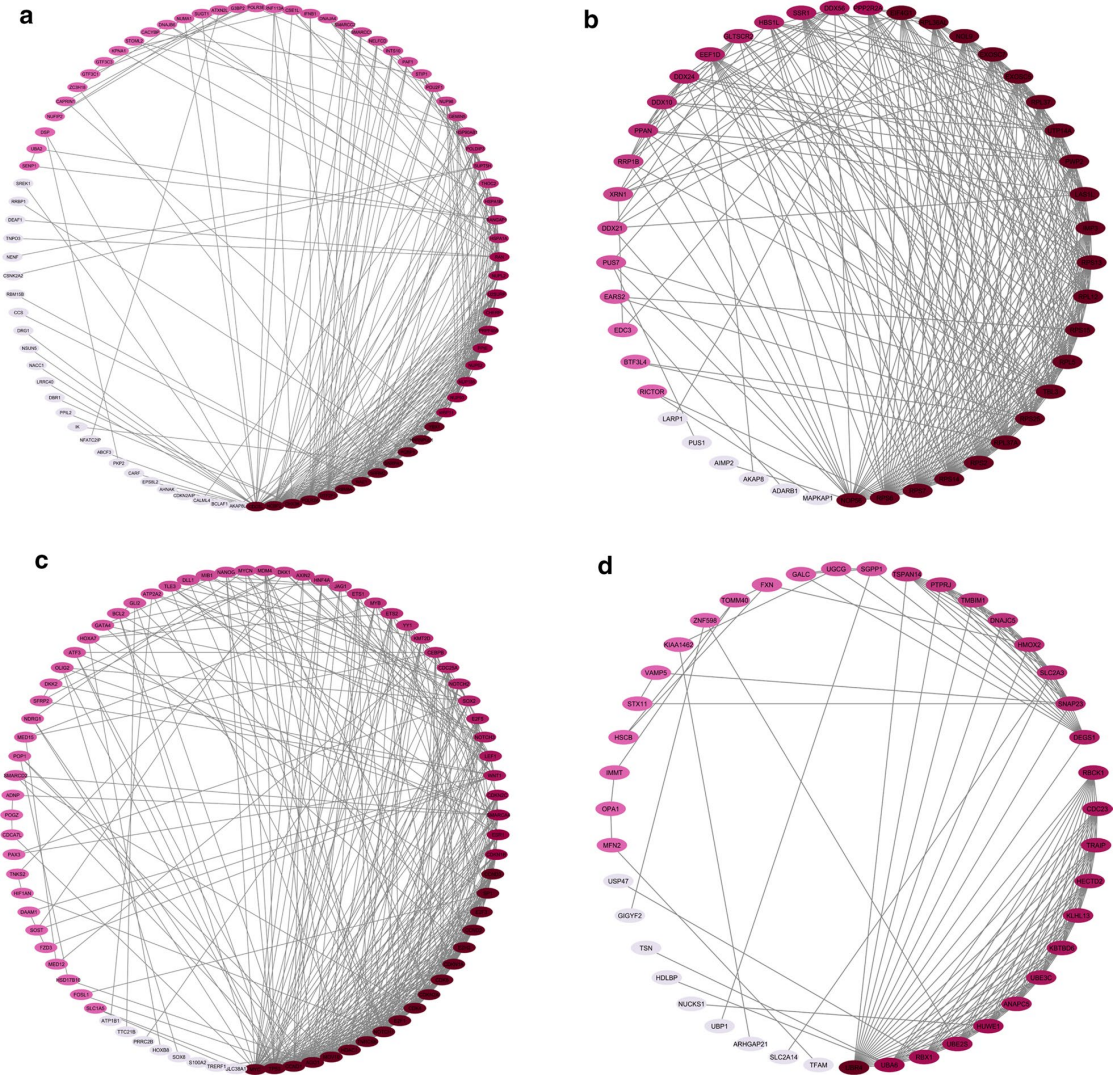
The PPINs constituted between the identified hub DEGs **a** Module 1, **b** Module 2, **c** Module 3, and **d** Module 4 of Normal vs. ACs group. The color is indicative of the degree level, so that bold color indicates the higher degree of node

To explore more information about the relationships between the DEGs, PPINs were constructed by STRING. The networks were analyzed in terms of topology and centrality parameters. The nodes with a higher degree and betweenness were selected as hub genes. From these analyses, 24 and 6 hub genes were specified for normal vs. HAM/TSP and ACs vs. HAM/TSP groups, respectively (Fig. 1a, b). The highly connected network of the Normal vs. AC group caused that the modules were explored. A total of 23 modules were identified, which four of them including 251 genes were highly connected and biologically meaningful (Fig. 2a–d).

The color of each node in network is representative of the degree level from bold to pale color, which in turn shows the important role of that node in the network.

### Pathway enrichment

In order to find the biological pathway controlled by nodes of each network, the enrichment analysis was carried out. The modules identified from Normal vs. AC group enriched in the following pathways: Module 1: Metabolism of RNA, mRNA Splicing, RNA transport, HIV Infection, Rev-mediated nuclear export of HIV RNA, Infectious disease, Viral Messenger RNA Synthesis, and mRNA Processing; Module 2: rRNA processing, Metabolism of RNA, Viral mRNA Translation, Infectious disease, and Ribosome biogenesis in eukaryotes; Module 3: MicroRNAs in cancer, RNA Polymerase II Transcription, Pathways in cancer, Cell cycle, Signaling by NOTCH, Regulation of RUNX1 Expression and Activity, p53 signaling pathway, Human T-cell leukemia virus 1 infection, Transcriptional regulation by RUNX1, and Transcriptional misregulation in cancer; Module 4: Ubiquitin mediated proteolysis, Class I MHC mediated antigen processing & presentation, Antigen processing: Ubiquitination & Proteasome degradation, Adaptive Immune System, and Immune System. The nodes of Normal vs TSP group were enriched in Interferon Signaling, Cytokine Signaling in Immune system, Interferon alpha/beta signaling, Immune System, Interferon gamma signaling, JAK-STAT signaling pathway, Interleukin-6 family signaling, and Signaling by Interleukins. Finally, the following pathways were identified by enrichment of AC vs TSP group’s nodes: Transcriptional regulation by RUNX2 and Regulation of RUNX2 expression and activity (Table 2).

**Table 2:** The biological pathway which the hub genes in each group were enriched

normal vs. ACs	
Module 1	Metabolism of RNA, mRNA splicing, RNA transport, HIV Infection, Rev-mediated nuclear export of HIV RNA, Infectious disease, viral messenger RNA synthesis, and mRNA processing
Module 2	rRNA processing, Metabolism of RNA, Viral mRNA Translation, Infectious disease, and Ribosome biogenesis in eukaryotes
Module 3	MicroRNAs in cancer, RNA polymerase II transcription, pathways in cancer, Cell cycle, signaling by NOTCH, regulation of RUNX1 expression and activity, p53 signaling pathway, human T-cell leukemia virus 1 infection, Transcriptional regulation by RUNX1, and transcriptional misregulation in cancer
Module 4	Ubiquitin mediated proteolysis, Class I MHC mediated antigen processing & presentation, antigen processing: ubiquitination & proteasome degradation, adaptive immune system, and immune system
normal vs. HAM/TSP	
Interferon signaling, cytokine signaling in immune system, interferon alpha/beta signaling, immune system, interferon gamma signaling, JAK-STAT signaling pathway, interleukin-6 family signaling, and signaling by interleukins	
ACs vs. HAM/TSP	
Transcriptional regulation by RUNX2 and regulation of RUNX2 expression and activity	

### Demographic data

The mean age of three groups was as follow: normal controls: 41 ± 2.8, ACs: 42 ± 3.5, and HAM/TSP patients: 48 ± 3.6. Any significant difference was found between the ages of three groups.

### Flow cytometry

**Fig. 3:**
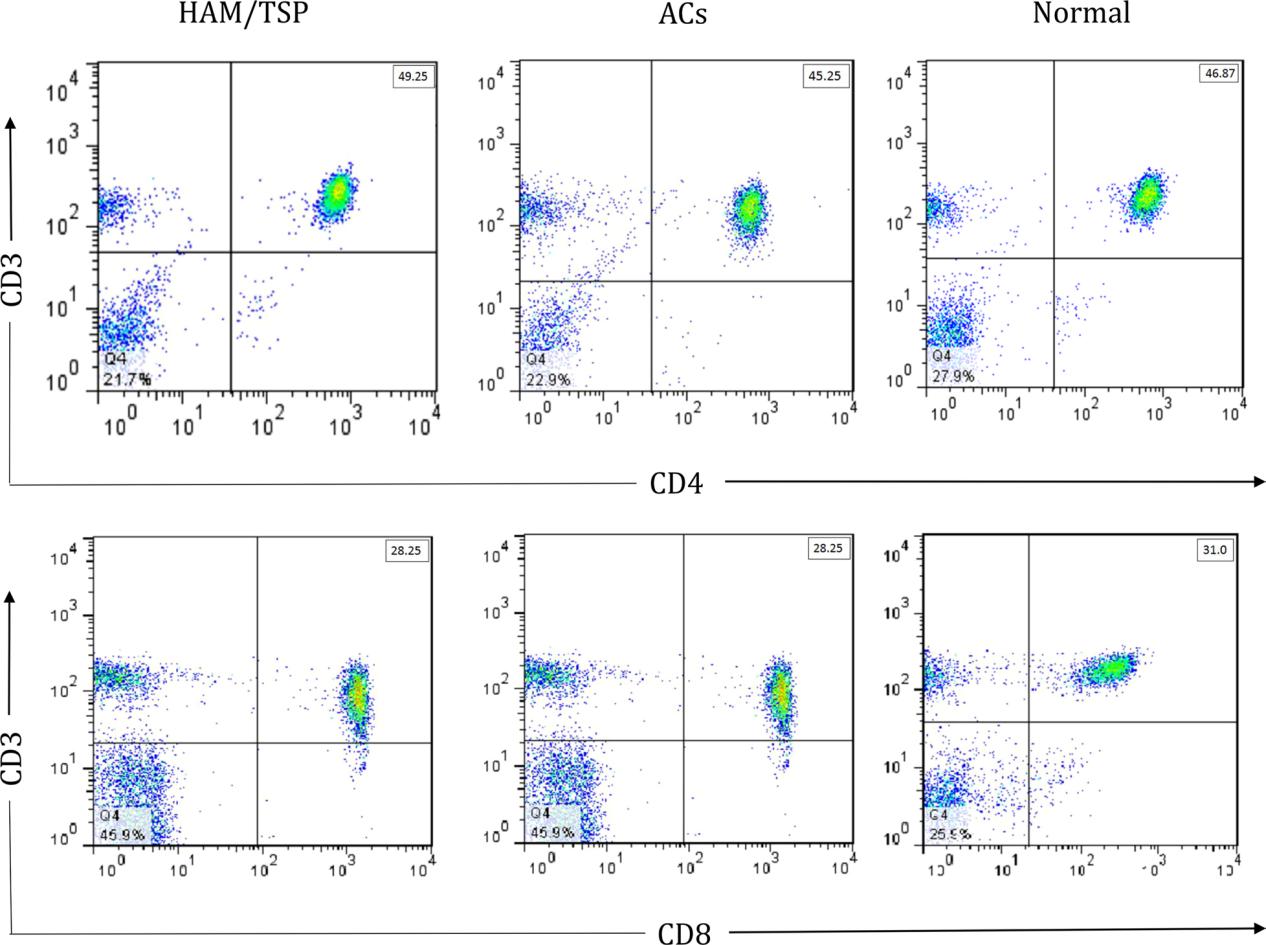
Flow cytometry data analyze of T helper and cytotoxic T LYMPHOCYTES

Flow Cytometry Data Analyze of T helper and cytotoxic T lymphocytes was done by Flowjo 7.6.1. No significant difference was found among the three groups in terms of the percentage of T helper (*P *= 0.55) and cytotoxic T lymphocytes (*P *= 0.12) (Fig. 3).

### HTLV-1 proviral load

**Fig. 4:**
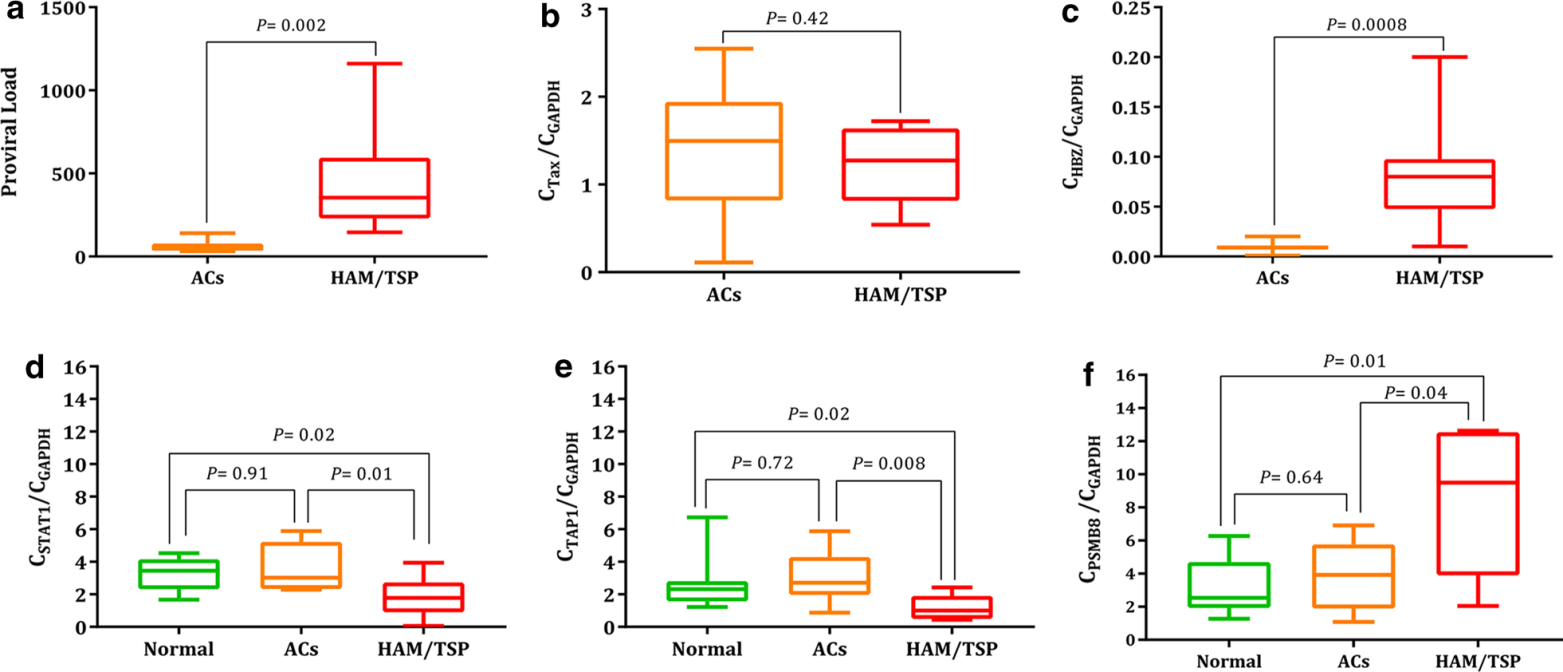
**a** HTLV-I- proviral load. The PVL in HAM/TSP patients was significantly higher than in ACs (*P* = 0.002). **b** Tax gene expression. No significant difference was found between ACs and HAM/TSP groups (*P* = 0.42). **c** HBZ gene expressions which was significantly higher in the HAM/TSP group than that in the ACs group (*P* = 0.0008). **d** STAT1 gene expressions in the Normal, ACs, and HAM/TSP groups. STAT1 gene expression in the HAM/TSP was significantly higher than in Normal (*P* = 0.02). The STAT1 between AC and HAM/TSP patients was statistically different (*P* = 0.01). No significant difference was found between Normal and AC patients (*P* = 0.91). **e** TAP1 gene expressions in the Normal, ACs, and HAM/TSP groups. TAP1 gene expression in the HAM/TSP was significantly higher than in Normal (*P* = 0.02). The TAP1 between AC and HAM/TSP patients was statistically different (*P* = 0.008). No significant difference was found between Normal and AC patients (*P* = 0.72). **e** PSMB8 gene expressions in the Normal, ACs, and HAM/TSP groups. PSMB8 gene expression in the HAM/TSP was significantly higher than in Normal (*P* = 0.01). The PSMB8 between AC and HAM/TSP patients was statistically different (*P* = 0.04). No significant difference was found between Normal and AC patients (*P* = 0.64)

All HAM/TSP patients had proviral loads (PVLs) in the range of 216–1160 and all ACs had PVLs in the range of 32–140. The mean PVL of HTLV-1 in the HAM/TSP patients was 455.8 ± 114.7, which was significantly higher (*P *= 0.002) than that in the ACs (60.88 ± 12.92) (Fig. 4a).

### Real time-quantitative PCR for validation of expression changes

The expression levels of *Tax* and *HBZ* were measured in the samples, which revealed the insignificant up-regulation of *Tax* in ACs group (1.41 ± 0.27) than that in HAM/TSP (1.22 ± 0.16) group (*P *= 0.42) and significant higher expression level of *HBZ* in HAM/TSP group (0.08 ± 0.01) than that in ACs group (0.009 ± 0.001) (*P *= 0.0008) (Fig. 4b, c).

Moreover, the network analyses revealed STAT1 and PSMB8 as the nodes with high degree value in normal vs. TSP and ACs vs. TSP groups. Therefore, we examined them with TAP1 as a random gene for further step of validating the meta-analysis results. The differential expression of these genes was analyzed by comparing expression levels in PBMCs of normal, ACs, and HAM/TSP subjects using RT-qPCR. To this purpose, the differential expressions of genes were analyzed by comparing expression levels in normal, AC, and HAM/TSP samples. The results revealed the meaningful down-regulation of STAT1 in HAM/TSP (1.8 ± 0.43) samples than those in the AC (3.6 ± 0.52) and normal (3.3 ± 0.36) samples (*P *= 0.01 and *P *= 0.02, respectively) (Fig. 4d). The remarkable down-regulation of *TAP1* in HAM/TSP (1.2 ± 0.27) samples than those in the AC (3.0 ± 0.56) and normal (2.7 ± 0.61) samples was observed (*P *= 0.008 and *P *= 0.02, respectively) (Fig. 4e). Also, the expression level of *PSMB8* has significantly increased in the HAM/TSP (8.5 ± 1.5) samples than those in the AC (3.8 ± 0.74) and normal (3.1 ± 0.61) samples (*P *= 0.04 and *P *= 0.01, respectively) (Fig. 4f). Moreover, the correlation analysis was done to determine the association between different factors. The results indicated the significant correlation between STAT1 and PVL (*P *= 0.04, r = 0.74) and also between *STAT1* and *PSMB8* (*P *= 0.03, r = 0.76) in ACs group. The remarkable associations were observed between Tax and TAP1 (*P *= 0.04, r = 0.73), *STAT1* and PSMB8 (*P *= 0.02, r = 0.78), *HBZ* and PVL (*P *= 0.05, r = 0.70) in HAM/TSP group.

## Discussion

Despite four decades of researches on HTLV-1, many questions remain regarding the pathogenicity mechanism and key proteins involved in various pathological pathways. Moreover, it is also ambiguous which factors and proteins determine the final destiny of infection by HTLV1 toward HAM/TSP or/and ATLL, while some infected subjects remain in the form of asymptomatic carriers.

Microarray technology is widely used to analyze and measure gene expression at the high-throughput scale. Despite the high benefits of using this technology, the result of a population cannot be generalized to another population. Data integration and providing a meta-analysis of the reported data improve the validity and reliability of the results. Genomics, transcriptomics, and proteomics data can be combined to find biomarkers and possible pathogenesis pathways [23].

From differential expression analysis of the miRNAs samples between normal and ACs groups, four miRNAs including hsa-mir-218, hsa-mir-206, hsa-mir-31, and hsa-mir-34A were identified, which can be considered as biomarkers for diagnosis of AC state.

In complying with previous reports, the identified DEGs were involved in the immune system of the HAM/TSP subjects. Moreover, the involved molecular network as the primary model was introduced through collection and integration of high-throughput data. We validated two main hub genes of *STAT1* and *PSMB8,* and also *TAP1* to confirm our results.

STAT1 is an important intermediary in responding to IFNs. After binding IFN-I to the cellular receptor, signal transduction occurs through protein kinases which results in the activation of Jak kinase. It, in turn, causes phosphorylation of tyrosine in STAT1 and STAT2. The activated STATs are embedded in the dimer with ISGF3 and IRF9 and enter the nucleus which leads to up-regulation of IFNs and enhances the antiviral response [41, 42]. The significant down-regulation of STAT1 in patients with HAM/TSP was observed compared with asymptomatic carriers and healthy individuals. The decrease in the expression of STAT1 is the response of the infected cells to escape HTLV-1 from the immune response associated with HAM/TSP.

The expression change of STAT1 in ATLL patients has been reported in several studies [43]. However, no studies have addressed the dysregulation of STAT1 expression in HAM/TSP patients. The reduction of STAT1 and subsequent MHC-I in this disease can significantly affect the action of CD8 and NK cells as important cells in the HAM/TSP pathogenesis [44, 45].

A significant increase was observed in the expression of PSMB8 in patients with HAM/TSP in comparison to those who carry the virus and normal subjects. PSMB8 is one of the 17 subunits essential for the synthesis of the 20S proteasome unit [46]. The targeting of proteasome in the HAM/TSP disease is a known mechanism which affects the pathogenicity of HTLV-1 by increasing the activity of genes such as IKBKG [2]. PSMB8 can influence the immune responses due to involvement in the process of apoptosis [47], so its increase in patients with HAM/TSP may be because of this function. Although previous studies reported the role of apoptosis in the HAM/TSP pathogenesis [2], there is no comprehensive information regarding the role of PSMB8.

TAP1 is another gene which significantly down-regulated in the HAM/TSP group compared with asymptomatic carriers and normal groups. TAP1 protein which is expressed by the TAP gene involves the transfer of antigen from the cytoplasm to the endoplasmic reticulum to accompany with MHC-I. HTLV-1 seems to run out from the antiviral response in association with MHC-I due to impairment in the TAP1 function [48]. Such occurrence was also observed as a result of infections by other viruses such as EBV, CMV, and adenovirus [49]. Similar to STAT1, a

It is noteworthy that the immune decrease in the TAP1 expression can also significantly affect CD8 and NK cells [44, 45]. Therefore, it seems that escaping from CTL-immune response is one of the important mechanisms for pathogenicity in HAM/TSP; however, more accurate and detailed studies are needed. In HAM/TSP, the disorder expression of the STAT1 and TAP1 proteins can disrupt the immune system.

In HAM/TSP disease, PSMB8 in associated with PSMB8, JAK2, STAT1, IFI35, IRF1, GBP2, IFITM1, HLA-B, ISG20, GBP5, SOCS1, BST2, IFNG, and UBE2L6 activate the Interferon Signaling pathway and Cytokine Signaling in Immune system. The importance of cytokines especially IFN-γ for the HAM ⁄TSP pathogenesis were previously reported [50, 51].

The enrichment of modules identified from normal vs. ACs group revealed the involvement of hub genes in Infectious disease, Viral Messenger RNA Synthesis, Metabolism of RNA, Pathways in Cancer, Human T-cell leukemia virus 1 infection, and Antigen processing which activate after virus infection and asymptomatic state. These hub genes can be more evaluated in further studies.

The mechanisms involved in the HAM/TSP development are complicated, so identification of proteins which have different expressions than the normal group is critical to find the complete pathogenesis pathway [2].

Determining viral factors such as proviral load along with measuring the expression levels of Tax and HBZ genes will be effective in finding the virus action in the patient group. Moreover, host-related factors such as age, family history of the disease, genetics, and host immune status are important [52–57].

Destruction of cells in the central nervous system may be due to the release of inflammatory substances from lymphocytes produced by the immune response to the contaminated TCD4+ cells, which are referred as “bystander” damage. It is most likely the mechanism of tissue damage in HAM/TSP disease. In this study, there was no significant difference in the ratio of CD4 to CD8 in the HAM/TSP patients than asymptomatic carriers and healthy subjects; however, a slight increase was observed in the asymptomatic carriers group in comparison to the HAM/TSP and healthy subjects. This may be due to the function of the immunity system to prevent virus replication and progress toward HAM/TSP disease, but more studies with a higher sample size are required. Eventually, patients with HAM/TSP have impairment in their immune system induced by the HTLV-1 infection, which includes the innate and adaptive immunity to develop the disease and increase apoptosis [2].

## Conclusion

We employed meta-analysis of high throughput data to find the involved genes in the pathogenesis mechanisms of HAM/TSP disease. The network analysis disclosed novel hub genes involved in important pathways in virus infection and then interferon, cytokine, interleukin, and immune systems. Finally, the comprehensive studies are needed to improve our knowledge about the pathogenesis pathways and also biomarkers of complex diseases.

## Supplementary information

**Additional file 1: Table S1.** List of the identified DEGs in each group.

## Data Availability

All relevant data are within the paper.

## Abbreviations

HTLV-1: human T-cell leukemia virus type 1
AC: asymptomatic carrier
HAM/TSP: HTLV-1-associated myelopathy/tropical spastic paraparesis
ATLL: adult T cell leukemia/lymphoma
DEGs: differentially expressed genes
DEMs: differentially expressed miRNAs
PBMCs: peripheral blood mononuclear cells
PPINs: protein–protein interactions networks

## References

[1] Andrade RG, Goncalves Pde C, Ribeiro MA, Romanelli LC, Ribas JG, Torres EB, Carneiro-Proietti AB, Barbosa-Stancioli EF, Martins ML (2013). Strong correlation between tax and HBZ mRNA expression in HAM/TSP patients: distinct markers for the neurologic disease. J Clin Virol.

[2] Mozhgani SH, Zarei-Ghobadi M, Teymoori-Rad M, Mokhtari-Azad T, Mirzaie M, Sheikhi M, Jazayeri SM, Shahbahrami R, Ghourchian H, Jafari M (2018). Human T-lymphotropic virus 1 (HTLV-1) pathogenesis: a systems virology study. J Cell Biochem.

[3] Trevino A, Aguilera A, Caballero E, Benito R, Parra P, Eiros JM, Hernandez A, Calderon E, Rodriguez M, Torres A (2012). Trends in the prevalence and distribution of HTLV-1 and HTLV-2 infections in Spain. Virol J.

[4] Ahmadi Ghezeldasht S, Shirdel A, Assarehzadegan MA, Hassannia T, Rahimi H, Miri R, Rezaee SA (2013). Human T lymphotropic virus type I (HTLV-I) oncogenesis: molecular aspects of virus and host interactions in pathogenesis of adult T cell leukemia/lymphoma (ATL). Iran J Basic Med Sci.

[5] Shoeibi A, Etemadi M, Moghaddam Ahmadi A, Amini M, Boostani R (2013). “HTLV-I Infection” twenty-year research in neurology Department of Mashhad University of Medical Sciences. Iran J Basic Med Sci.

[6] Nakagawa M, Izumo S, Ijichi S, Kubota H, Arimura K, Kawabata M, Osame M (1995). HTLV-I-associated myelopathy: analysis of 213 patients based on clinical features and laboratory findings. J Neurovirol.

[7] Kchour G, Rezaee R, Farid R, Ghantous A, Rafatpanah H, Tarhini M, Kooshyar MM, El Hajj H, Berry F, Mortada M (2013). The combination of arsenic, interferon-alpha, and zidovudine restores an “immunocompetent-like” cytokine expression profile in patients with adult T-cell leukemia lymphoma. Retrovirology.

[8] Hermine O, Wattel E, Gessain A, Bazarbachi A (1998). Adult T cell leukaemia: a review of established and new treatments. BioDrugs.

[9] Olindo S, Belrose G, Gillet N, Rodriguez S, Boxus M, Verlaeten O, Asquith B, Bangham C, Signaté A, Smadja D (2011). Safety of long-term treatment of HAM/TSP patients with valproic acid. Blood.

[10] Nakamura T (2009). HTLV-I-associated myelopathy/tropical spastic paraparesis (HAM/TSP): the role of HTLV-I-infected Th1 cells in the pathogenesis, and therapeutic strategy. Folia Neuropathol.

[11] Oh U, Jacobson S (2008). Treatment of HTLV-I-associated myelopathy/tropical spastic paraparesis: toward rational targeted therapy. Neurol Clin.

[12] Tagaya Y (2019). A-109 A novel multi-cytokine inhibitory strategy in treating HTLV-1 diseases. JAIDS J Acquir Immune Defic Syndr.

[13] Moriuchi M, Moriuchi H, Fauci AS (1999). Htlv-i tax activation of the Cxcr4 promoter by association with nuclear respiratory factor 1. JAIDS J Acquir Immune Defic Syndr.

[14] Ottoson NC, Pribila JT, Chan AS, Shimizu Y (2001). Cutting edge: T cell migration regulated by CXCR4 chemokine receptor signaling to ZAP-70 tyrosine kinase. J Immunol.

[15] Schena M, Shalon D, Davis RW, Brown PO (1995). Quantitative monitoring of gene expression patterns with a complementary DNA microarray. Science.

[16] Ntzani EE, Ioannidis JP (2003). Predictive ability of DNA microarrays for cancer outcomes and correlates: an empirical assessment. Lancet.

[17] Michiels S, Koscielny S, Hill C (2005). Prediction of cancer outcome with microarrays: a multiple random validation strategy. Lancet.

[18] Ein-Dor L, Kela I, Getz G, Givol D, Domany E (2005). Outcome signature genes in breast cancer: is there a unique set?. Bioinformatics.

[19] Li S, Rouphael N, Duraisingham S, Romero-Steiner S, Presnell S, Davis C, Schmidt DS, Johnson SE, Milton A, Rajam G (2014). Molecular signatures of antibody responses derived from a systems biology study of five human vaccines. Nat Immunol.

[20] Pena OM, Hancock DG, Lyle NH, Linder A, Russell JA, Xia J, Fjell CD, Boyd JH, Hancock RE (2014). An endotoxin tolerance signature predicts sepsis and organ dysfunction at initial clinical presentation. EBioMedicine.

[21] Zhang G, He P, Tan H, Budhu A, Gaedcke J, Ghadimi BM, Ried T, Yfantis HG, Lee DH, Maitra A (2013). Integration of metabolomics and transcriptomics revealed a fatty acid network exerting growth inhibitory effects in human pancreatic cancer. Clin Cancer Res.

[22] Gieger C, Geistlinger L, Altmaier E, Hrabe de Angelis M, Kronenberg F, Meitinger T, Mewes HW, Wichmann HE, Weinberger KM, Adamski J (2008). Genetics meets metabolomics: a genome-wide association study of metabolite profiles in human serum. PLoS Genet.

[23] Ramasamy A, Mondry A, Holmes CC, Altman DG (2008). Key issues in conducting a meta-analysis of gene expression microarray datasets. PLoS Med.

[24] Kang DD, Sibille E, Kaminski N, Tseng GC (2012). MetaQC: objective quality control and inclusion/exclusion criteria for genomic meta-analysis. Nucleic Acids Res.

[25] Huang HY, Lin YCD, Li J, Huang KY, Shrestha S, Hong HC, Tang Y, Chen YG, Jin CN, Yu Y (2019). miRTarBase 2020: updates to the experimentally validated microRNA–target interaction database. Nucleic Acids Res.

[26] Szklarczyk D, Morris JH, Cook H, Kuhn M, Wyder S, Simonovic M, Santos A, Doncheva NT, Roth A, Bork P (2016). The STRING database in 2017: quality-controlled protein–protein association networks, made broadly accessible. Nucleic Acids Res.

[27] Koen EL, Bowman J, Wilson PJ (2016). Node-based measures of connectivity in genetic networks. Mol Ecol Resour.

[28] Rezadoost H, Karimi M, Jafari M (2016). Proteomics of hot-wet and cold-dry temperaments proposed in Iranian traditional medicine: a Network-based Study. Sci Rep.

[29] Bastian M, Heymann S, Jacomy M (2009). Gephi: an open source software for exploring and manipulating networks. Icwsm.

[30] Reimand J, Isserlin R, Voisin V, Kucera M, Tannus-Lopes C, Rostamianfar A, Wadi L, Meyer M, Wong J, Xu C (2019). Pathway enrichment analysis and visualization of omics data using g: profiler, GSEA, Cytoscape and EnrichmentMap. Nat Protoc.

[31] Rafatpanah H, Torkamani M, Valizadeh N, Vakili R, Meshkani B, Khademi H, Gerayli S, Mozhgani SH, Rezaee SA (2016). Prevalence and phylogenetic analysis of HTLV-1 in a segregated population in Iran. J Med Virol.

[32] Boostani R, Vakili R, Hosseiny SS, Shoeibi A, Fazeli B, Etemadi MM, Sabet F, Valizade N, Rezaee SA (2015). Triple therapy with prednisolone, pegylated interferon and sodium valproate improves clinical outcome and reduces human T-cell leukemia virus type 1 (HTLV-1) proviral load, tax and HBZ mRNA expression in patients with HTLV-1-associated myelopathy/tropical spastic paraparesis. Neurotherapeutics.

[33] Mozhgani SH, Jahantigh HR, Rafatpanah H, Valizadeh N, Mohammadi A, Basharkhah S, Rezaee SA (2018). Interferon lambda family along with HTLV-1 proviral load, tax, and HBZ implicated in the pathogenesis of myelopathy/tropical spastic paraparesis. Neurodegener Dis.

[34] Jafarian M, Mozhgani SH, Patrad E, Vaziri H, Rezaee SA, Akbarin MM, Norouzi M (2017). Evaluation of INOS, ICAM-1, and VCAM-1 gene expression: a study of adult T cell leukemia malignancy associated with HTLV-1. Arch Virol.

[35] Tattermusch S, Skinner JA, Chaussabel D, Banchereau J, Berry MP, McNab FW, O’Garra A, Taylor GP, Bangham CR (2012). Systems biology approaches reveal a specific interferon-inducible signature in HTLV-1 associated myelopathy. PLoS Pathog.

[36] Vernin C, Thenoz M, Pinatel C, Gessain A, Gout O, Delfau-Larue M-H, Nazaret N, Legras-Lachuer C, Wattel E, Mortreux F (2014). HTLV-1 bZIP factor HBZ promotes cell proliferation and genetic instability by activating OncomiRs. Cancer Res.

[37] Thénoz M, Vernin C, Mortada H, Karam M, Pinatel C, Gessain A, Webb TR, Auboeuf D, Wattel E, Mortreux F (2014). HTLV-1-infected CD4+ T-cells display alternative exon usages that culminate in adult T-cell leukemia. Retrovirology.

[38] Kobayashi S, Nakano K, Watanabe E, Ishigaki T, Ohno N, Yuji K, Oyaizu N, Asanuma S, Yamagishi M, Yamochi T (2014). CADM1 expression and stepwise downregulation of CD7 are closely associated with clonal expansion of HTLV-I—infected cells in adult T-cell leukemia/lymphoma. Clin Cancer Res.

[39] Yeung ML, Yasunaga J-I, Bennasser Y, Dusetti N, Harris D, Ahmad N, Matsuoka M, Jeang K-T (2008). Roles for microRNAs, miR-93 and miR-130b, and tumor protein 53—induced nuclear protein 1 tumor suppressor in cell growth dysregulation by human T-cell lymphotrophic virus 1. Cancer Res.

[40] Pinto MT, Malta TM, Rodrigues ES, Pinheiro DG, Panepucci RA, Farias K, de Paula Alves Sousa A, Takayanagui O, Tanaka Y, Covas DT (2013). Genes related to antiviral activity, cell migration, and lysis are differentially expressed in CD4+ T cells in HAM/TSP patients. AIDS Res Hum Retrovir.

[41] Chen K, Liu J, Liu S, Xia M, Zhang X, Han D, Jiang Y, Wang C, Cao X (2017). Methyltransferase SETD2-mediated methylation of STAT1 is critical for interferon antiviral activity. Cell.

[42] Zhang Y, Mao D, Roswit WT, Jin X, Patel AC (2015). PARP9-DTX3L ubiquitin ligase targets host histone H2BJ and viral 3C protease to enhance interferon signaling and control viral infection. Nat Immunol.

[43] Moles R, Bellon M, Nicot C (2015). STAT1: a novel target of miR-150 and miR-223 is involved in the proliferation of HTLV-I-transformed and ATL cells. Neoplasia.

[44] Saito M (2010). Immunogenetics and the pathological mechanisms of human T-cell leukemia virustype 1-(HTLV-1-)associated myelopathy/tropical spastic paraparesis (HAM/TSP). Interdiscip Perspect Infect Dis.

[45] Yu F, Itoyama Y, Fujihara K, Goto I (1991). Natural killer (NK) cells in HTLV-I-associated myelopathy/tropical spastic paraparesis-decrease in NK cell subset populations and activity in HTLV-I seropositive individuals. J Neuroimmunol.

[46] Akiyama K, Kagawa S, Tamura T, Shimbara N, Takashina M, Kristensen P, Hendil KB, Tanaka K, Ichihara A (1994). Replacement of proteasome subunits X and Y by LMP7 and LMP2 induced by interferon-gamma for acquirement of the functional diversity responsible for antigen processing. FEBS Lett.

[47] Yang Z, Gagarin D, St Laurent G, Hammell N, Toma I, Hu CA, Iwasa A, McCaffrey TA (2009). Cardiovascular inflammation and lesion cell apoptosis: a novel connection via the interferon-inducible immunoproteasome. Arterioscler Thromb Vasc Biol.

[48] Bahram S, Arnold D, Bresnahan M, Strominger JL, Spies T (1991). Two putative subunits of a peptide pump encoded in the human major histocompatibility complex class II region. Proc Natl Acad Sci.

[49] Verweij MC, Horst D, Griffin BD, Luteijn RD, Davison AJ, Ressing ME, Wiertz EJHJ (2015). Viral inhibition of the transporter associated with antigen processing (TAP): a striking example of functional convergent evolution. PLoS Pathog.

[50] Montanheiro P, Penalva de Oliveira A, Smid J, Fukumori L, Olah I, Duarte ADS, Casseb J (2009). The elevated interferon gamma production is an important immunological marker in HAM/TSP pathogenesis. Scand J Immunol.

[51] Muniz AL, Rodrigues W, Santos SB, Jesus ARD, Porto AF, Castro N, Oliveira-Filho J, Almeida JP, Moreno-Carvalho O, Carvalho EM (2006). Association of cytokines, neurological disability, and disease duration in HAM/TSP patients. Arq Neuropsiquiatr.

[52] Akbarin MM, Rahimi H, Hassannia T, Shoja Razavi G, Sabet F, Shirdel A (2013). Comparison of HTLV-I proviral load in adult T cell leukemia/lymphoma (ATL), HTLV-I-associated myelopathy (HAM-TSP) and healthy carriers. Iran J Basic Med Sci.

[53] Asquith B, Bangham CR (2008). How does HTLV-I persist despite a strong cell-mediated immune response?. Trends Immunol.

[54] Iwanaga M, Watanabe T, Utsunomiya A, Okayama A, Uchimaru K, Koh KR, Ogata M, Kikuchi H, Sagara Y, Uozumi K (2010). Human T-cell leukemia virus type I (HTLV-1) proviral load and disease progression in asymptomatic HTLV-1 carriers: a nationwide prospective study in Japan. Blood.

[55] Matsuzaki T, Nakagawa M, Nagai M, Usuku K, Higuchi I, Arimura K, Kubota H, Izumo S, Akiba S, Osame M (2001). HTLV-I proviral load correlates with progression of motor disability in HAM/TSP: analysis of 239 HAM/TSP patients including 64 patients followed up for 10 years. J Neurovirol.

[56] Nagai M, Usuku K, Matsumoto W, Kodama D, Takenouchi N, Moritoyo T, Hashiguchi S, Ichinose M, Bangham CR, Izumo S, Osame M (1998). Analysis of HTLV-I proviral load in 202 HAM/TSP patients and 243 asymptomatic HTLV-I carriers: high proviral load strongly predisposes to HAM/TSP. J Neurovirol.

[57] Okayama A, Stuver S, Matsuoka M, Ishizaki J, Tanaka G, Kubuki Y, Mueller N, Hsieh CC, Tachibana N, Tsubouchi H (2004). Role of HTLV-1 proviral DNA load and clonality in the development of adult T-cell leukemia/lymphoma in asymptomatic carriers. Int J Cancer.

